# Supplementary material for: WordCommentsAnalyzer: A windows software tool for qualitative research
Source: F1000Res. 2018 Sep 4;7:536. Originally published 2018 May 3. [Version 2] doi: 10.12688/f1000research.14819.2 (PMC6137411; doi:10.12688/f1000research.14819.2)
Supplement: Supplementary file 3 [file f1000research-7-17614-s0002.tgz › b2a79496-4ada-47c4-9682-a99ce867836d.zip!/Eight Iranian Health Journals/Journal of Health System Research _30.docx]

© Journal of Health System Research . This work is licensed under CC BY-NC 4.0 (https://creativecommons.org/licenses/by-nc/4.0/).

Volume & Issue: دوره 9، شماره 2؛ 1392

Title: رویکرد سلامت در همه سیاست‌ها

http://hsr.mui.ac.ir/index.php/jhsr/article/view/507

پیاده‌سازی رویکرد «سلامت در همه سیاست‌ها» (HiAP یا Health in all polices) در یک سیستم، دولت را قادر می‌‌سازد تا در پاسخ‌دهی به نیازهای سلامت و تندرستی مردم یک جامعه یکپارچه عمل کند. رویکرد «سلامت در همه سیاست‌ها» اثرات سایر خط و مشی‌ها و قوانین بر روی سلامت را از طریق تعیین کننده‌های سلامت در نظر می‌گیرد (1). تعیین کننده‌های سلامت می‌توانند به طور مستقیم و آنی از سیاست‌ها و مداخلات در بخش‌های مختلف متأثر شوند. رویکرد سلامت در همه سیاست‌ها وقتی تحقق می‌یابد که قوانین و سیاست‌های سلامت محور در همه بخش‌ها طراحی، برنامه‌ریزی و اجرا شود. این رویکرد همچنین اثرات سیاست‌های موجود را نیز ارزیابی می‌کند و هدف نهایی‌ آن، افزایش سیاست‌گذاری مبتنی بر اطلاعات (Evidence informed policy-making)، آگاه ساختن سیاست‌گذاران با ایجاد پیوند بین سیاست‌ها و مداخلات، تعیین کننده‌های سلامت و پیامدها و نتایج سلامت است؛ به طوری که هدف نهایی این رویکرد از میان برداشتن شکاف نابرابری سلامت می‌باشد (2). سیاست‌ها می‌توانند دارای کارکرد مثبت و منفی بر تعیین کننده‌های سلامت باشند. برای مثال، سلامت قلب به دنبال ایجاد تغییراتی در سیاست‌ها و قانون‌گذاری در سایر بخش‌‌ها اعم از سازمان‌های متولی امر سلامت و غیر آن صورت گرفته است که موجب افزایش کیفیت غذا، کاهش استعمال دخانیات و سیگار و افزایش فعالیت بدنی گردیده است. نمونه‌هایی از تأثیرات سایر سیاست‌ها بر سلامت در جدول 1 آمده است. نکته مهم و اساسی این است که بدانیم سیاست‌های تعیین کننده سلامت به هیچ عنوان دارای مفهوم خنثی نیستند (3). به دنبال چالش‌های اجتماعی غیر قابل اجتناب یکپارچه‌سازی، سیاست‌ها و خط و مشی‌ها بیش از گذشته دارای اهمیت است. بسیاری از سیاست‌های ملی در بخش تجارت و صنعت، بازارهای داخلی و کشاورزی در جهت سیاست‌های بین‌المللی رشد خواهند کرد. در کل جهت رشد سیاست‌ها از سطح ملی به سطح بین‌المللی است، اما گاهی این رابطه معکوس خواهد شد و تحت فشارهای جهانی لازم است که سیاست‌های بین‌المللی در سطوح ملی مورد بررسی و اقدام قرار گیرند (4). دو استراتژی و راهبرد در پیاده‌سازی این رویکرد تعریف شده است. در حالت اول، مواردی که سود دو جانبه بین بخش بهداشت و سایر سازمان‌ها وجود داشته باشد، ترکیب اهداف به راحتی امکان‌پذیر است و شرایط «برنده- برنده» (Win-win) ایجاد خواهد شد. در حالت دوم، وقتی که اهداف سلامت با اهداف پایه و اولیه سایر سیاست‌ها مطابقت نمی‌کند، در این صورت در نظر گرفتن اهداف سلامت به عنوان اهداف اصلی و پایه در اولویت قرار دارد؛ در این صورت یک راهبرد تک محوری وجود خواهد داشت (5). دموکراسی (Democracy)، مشارکت عمومی (Public participation) و شفاف‌سازی سیاست‌گذاری (Policy transparency) سه پیش‌نیاز مهم برای ارزش‌گذاری مفهوم سلامت در مکانیسم سیاست‌گذاری سلامت در سایر سازمان‌ها است. پیش‌نیازهای مذکور که در فرایند سیاست‌گذاری سایر سیاست‌ها نه فقط سیاست‌های سلامت محور گنجانده شده است، شامل شفاف‌سازی فرایند سیاست‌ها با توجه به محدوده زمانی معلوم، برقراری یک مذاکره و ارتباط خوب با متولیان سایر سیاست‌ها، آگاهی از دانش بهداشتی کافی و همچنین در نظر گرفتن سیاست‌های انتخابی و جایگزین است. بنابراین داشتن اطلاعات بهداشتی در هر گونه سیاست‌گذاری مبتنی بر شواهد ضروری است. این آگاهی باید بتواند کمک کند که سیاست اتخاذ شده روی سلامت چه تأثیری دارد؟ (16). سلامت یک مفهوم مثبت است که بر منابع اجتماعی و شخصی و همچنین ظرفیت‌های فیزیکی تأکید می‌کند. اگرچه نقش بخش سلامت به عنوان بخش فعال و حمایت کننده در همکاری‌های بین‌بخشی است و باید بیشترین تلاش‌ها برای تقویت همکاری با سایر سازمان‌ها در بخش بهداشت مدنظر قرار گیرد (1)، اما ارتقای سلامت نه فقط مسؤولیت‌بخش بهداشت است، بلکه فراتر از شیوه زندگی سالم که فقط نقش مردم را می‌بیند به سوی توسعه محیط حمایت کننده از سلامت مردم حرکت می‌کند. تحقیقات اخیر نشان داده است که عوامل بسیاری خارج از مراقبت‌های بهداشتی تأثیر زیادی بر سلامت دارند. از سیاست‌های کشاورزی که غذای روی میز شام ما را تحت تأثیر قرار می‌دهد تا تصمیمات ملی زیست محیطی که ما را در معرض خطر ابتلا به بیماری قرار داده است، همه به طور مستقیم و غیر مستقیم در انتخاب شیوه زندگی ما چه سالم و یا ناسالم و غیر بهداشتی اثر دارد. بنابراین ضروری است که تأثیر هر سیاست و تصمیمی اعم از بزرگ و کوچک، شخصی و سیاسی بر سلامت بررسی شود. بدون هیچ مصالحه‌ای باید با تجزیه و تحلیل، ردپای هر سیاست را بر روی سلامتی کشف کرد. از سیاست‌های سیستم حمل و نقل و آموزش و پرورش گرفته تا انرژی و تجارت، بی‌شک بر روی سلامت جامعه اثرگذار است. البته باید توجه داشت که فقط سیاست‌های کلان در سطح بین‌المللی و ملی مدنظر نمی‌باشد و به طور قطع باید به سیاست‌های اتخاذ شده در سطح منطقه‌ای و محلی نیز توجه گردد. بدین معنی که گاهی   جدول 1: نمونه‌هایی از تأثیرات سایر سیاست‌ها بر سلامت اقتصاد و کار اقتصاد انعطاف‌پذیر و پویا به افراد سالم وابسته است. نیروی کار سالم تجربه‌ای سازنده‌تر، با میزان غیبت کمتر در محیط کار و قابلیت سازگاری با تغییرات محیط کار را دارا می‌باشد. اشتغال امن و پایدار، سلامتی همه مردم در گروه‌های مختلف اجتماعی بهبود می‌دهد. کشاورزی و غذا توجه به سلامت و تندرستی در تولید، ساخت و بازاریابی موجب افزایش اعتماد مصرف کنندگان، بازار پایدار، نوآوری و رشد می‌شود. غذاهای سالم و در دسترس برای سلامت و رشد در تمام مراحل زندگی دارای اهمیت است. سیاست‌های اقتصادی و اجتماعی حمایت کننده‌های صنعت کشاورزی، موجب ارتقای سلامت و تندرستی آن جامعه خواهد شد. زیرساخت‌ها، برنامه‌ریزی و حمل و نقل جامعه سالم قادر به سرمایه‌گذاری در جهت رشد و توسعه پایدار است و از برنامه‌ریزی بهینه برای جاده‌ها، حمل و نقل و مسکن، کالاها و خدمات پشتیبانی می‌کند و تخریب محیط زیست را کاهش می‌دهد. در نتیجه رفاه و تندرستی از طریق ایجاد جوامع امن بهبود می‌یابد. محیط زیست و پایداری اکوسیستم شیوه زندگی سالم (موارد مربوط به مواد غذایی، فعالیت بدنی و مصرف مواد مخدر) آثار مخرب دی‌اکسیدکربن را کاهش داده و اکوسیستم پایدار را ارتقا می‌دهد. مسکن‌سازی و خدمات اجتماعی تمرکز بر روی سیاست‌های سلامت محور، بر طراحی برنامه و ارایه خدمات با هدف افزایش مشارکت اجتماعی- اقتصادی در از بین بردن محرومیت اجتماعی تأثیر خواهد داشت. مسکن در دسترس و حمایت اجتماعی و خانوادگی کافی عمده‌ترین تعیین کننده‌های سلامت افراد و جوامع محروم را مخاطب قرار خواهد داد. فرهنگ و تعلق به سرزمین مادری سرزمین و فرهنگ می‌تواند از سلامت بومی حمایت کند. رفاه بومی و فرهنگی محدود به یک حس عمیق تعلق خاطر به سرزمین و کشور است که تعریفی از هویت، باورها، ارزش‌ها و میراث فرهنگی هر جامعه می‌باشد. ارتقای سلامت بومی موجب تقویت فرهنگ جوامع و بهبود مشارکت عمومی می‌گردد.   سیاست اتخاذ شده توسط یک مدیر مدرسه یا معلم یک کلاس است که می‌تواند بر سلامت جسمی، روانی، اجتماعی و معنوی دانش‌آموزان تأثیر مثبت یا منفی داشته باشد (7). بنابراین چالش اصلی، آگاه ساختن سایر بخش‌ها از اثرات تصمیماتشان بر سلامت و ادغام اهداف سلامت با سایر سیاست‌ها است. در مقاله حاضر سعی بر آن شد تا با ارایه اجمالی از تعریف و تبیین هدف نهایی «رویکرد سلامت در همه سیاست‌ها» اهمیت یکپارچه‌سازی و سیاست‌گذاری مبتنی بر اطلاعات و تأثیر سیاست بر سلامت را پررنگ نماییم. بنابراین دسترسی سیاست‌گذاران و مجریان قوانین به اطلاعات معتبر بهداشتی ضروری است. سلامت تنها بخشی از انواع استراتژی‌ها و سیاست‌های یک جامعه در جهت ارزش ساخت مفهوم سلامت در بستر یکپارچگی سیاست‌ها می‌باشد. بخشی از قسمت‌های این مقاله ترجمه بولتن «سلامت در همه سیاست‌ها» در اتحادیه اروپا است (1).

Title: سطح آدیپونکتین سرم و میزان چربی‌های پلاسما

http://hsr.mui.ac.ir/index.php/jhsr/article/view/679

آدیپونکتین از جمله هورمون‌های ترشح شده از بافت چربی است که غلظت آن ارتباط معکوسی با توده چربی بدن دارد. این هورمون، ویژگی‌های ضد التهابی و ضد آتروژنیک داشته و غلظت آن در سندرم متابولیک، بیماری‌های قلبی- عروقی و اختلالات لیپیدی کاهش می‌یابد. بنابراین هدف مطالعه حاضر، مروری بر شواهد موجود در زمینه ارتباط میان غلظت آدیپونکتین سرم و سطح لیپیدهای پلاسما بود. مطالعات با طراحی کارآزمایی بالینی، مقطعی و آینده‌نگر با استفاده از موتور جستجوی PubMed، بین سال‌های 2002 تا 2011 و نیز استفاده از کلید واژه‌های Dislipidemia، Lipid profile، High or low density lipoprotein، Total cholesterol، Adiponectin و Triglyceride و محدود کردن به عنوان و چکیده آغاز گردید و در کل 25 مقاله مورد بررسی قرار گرفت. آدیپونکتین سرم به طور مثبت غلظت High density lipoprotein (HDL) پلاسما را تنظیم می‌کند. ارتباط معکوس و قابل توجهی میان غلظت تری‌گلیسیرید پلاسما و آدیپونکتین سرم وجود دارد. به نظر می‌رسد ارتباط معکوس میان غلظت  Very-low-density lipoprotein (VLDL) و Low density lipoprotein (LDL) با آدیپونکتین پلاسما وجود داشته باشد. رسپتورهای 2R آدیپونکتین نیز نقش مهمی در متابولیسم VLDL و تری‌گلیسیرید ایفا می‌کنند. در اختلالات سطح لیپیدهای خون غلظت آدیپونکتین کاهش می‌یابد و آدیپونکتین پلاسما نقش مهمی در متابولیسم لیپیدهای خون بر عهده دارد.

Title: چربی لبنیات و شاخص‌های لیپیدی خون

http://hsr.mui.ac.ir/index.php/jhsr/article/view/475

آمارها تاکنون نشان داده‌اند که بیماری‌های قلبی- عروقی علت تقریبی نیمی از مرگ و میرها در کشورهای صنعتی هستند و مصرف بعضی از چربی‌ها باعث افزایش چربی‌های مضر خون می‌شوند. یکی از منابع چربی مصرفی روزانه برای همه رژیم‌های غذایی، لبنیات است. بنابراین هدف از مقاله حاضر، مروری بر ارتباط بین چربی لبنیات و پروفایل لیپیدی خون بود. پس از جستجو با کلید واژه‌های Blood lipid،Dairy fat ، Milk cream و Cholesterol در پایگاه PubMed، بیش از 200 مقاله به دست آمد که پس از محدود کردن آن به زبان انگلیسی، دسترسی به تمام متن و سال نشر مقاله بین سال‌های 2002 تا 2011، از بین مقاله‌ها 14 مقاله در این زمینه مورد بررسی قرار گرفت. در بعضی از مطالعات کوهورت نشان داده شده است که مصرف لبنیات چرب باعث افزایش خطر بیماری‌های قلبی- عروقی می‌شود، ولی در بعضی دیگر ارتباطی پیدا نشده است و یا حتی ارتباط معکوسی داشته‌اند؛ به طور مثال در مطالعه «بررسی اثر مصرف لبنیات بر شیوع سندرم متابولیک» به ارتباط معکوس بین آن‌ها اشاره شده است و در مطالعه‌ای که شیوع بسیار پایین بیماری‌های قلبی در افراد قبیله‌ای آفریقا را نشان می‌دهد، وجود شیر به عنوان جزیی مهم از رژیم آن‌ها بوده است. نتایج در رابطه با این موضوع متناقض هستند و باید بررسی‌های بیشتری صورت گیرد و بهتر است مطالعات آینده‌نگر در این زمینه انجام گیرد.

Title: بررسی فعالیت‌های پژوهشی اعضای هیأت علمی دانشگاه علوم پزشکی رفسنجان در طی سال‌های 85- 1375

http://hsr.mui.ac.ir/index.php/jhsr/article/view/480

مقدمه: با توجه به اهمیت علم و نقش والای پژوهش در تولید علم و همچنین توجه به این نکته که دانشگاه‌ها محل اصلی پژوهش در همه جای دنیا هستند، در این تحقیق وضعيت فعاليت‌هاي پژوهشي اعضای هيأت علمي دانشگاه علوم پزشكي رفسنجان در طي سال‌های 85- 1375 مورد بررسی قرار گرفت. روش‌ها: در مطالعه مقطعی حاضر، کلیه فعالیت‌های پژوهشی اعضای هیأت علمی اعم از تعداد طرح‌های تحقیقاتی مصوب، تعداد مقالات منتشر شده به زبان‌های فارسی و انگلیسی، تعداد پایان‌نامه‌ها، سال و محل انجام پژوهش و جنسیت نویسنده اول از طریق واحد پژوهشی دانشگاه جمع‌آوری شد. داده‌ها با استفاده از نرم‌افزار SPSS و روش‌های آماری توصیفی و تحلیلی مانند آزمون 2c و Fisher exact test تجزیه و تحلیل شدند. یافته‌ها: مجموع فعالیت‌های پژوهشی، 1145 مورد بود که شامل 322 پایان‌نامه پزشکی (1/28 درصد)، 198 پایان‌نامه دندان‌پزشکی (3/17 درصد)، 443 طرح تحقیقاتی مصوب (7/38 درصد)، 118 مقاله داخلی (3/10 درصد) و 64 مقاله خارجی (6/5 درصد) می‌شد. سال 1385 با 191 فعالیت (7/16 درصد) و سال 1376 با 33 فعالیت (9/2 درصد) به ترتیب بیشترین و کمترین سهم را در انجام فعالیت‌های پژوهشی داشتند. بین تعداد افراد مشارکت‌کننده در فعالیت‌های پژوهشی با سال انجام پژوهش، دانشکده، جنسیت نویسنده و نوع فعالیت پژوهشی ارتباط معنی‌داری وجود داشت. نتیجه‌گیری: با توجه به سیر صعودی فراوانی فعالیت‌های علمی و افزایش افراد مشارکت کننده در این فعالیت‌ها می‌توان به بهبود سطح پژوهش در دانشگاه علوم پزشکی رفسنجان امیدوار بود، ولی جا دارد توجه بیشتری به حضور زنان در عرصه تحقیقات شود. همچنین، در راستای افزایش فعالیت‌های پژوهشی به صورت گروهی بهتر است تسهیلات بیشتری برای این‌گونه فعالیت‌ها در نظر گرفته شود. واژه‌‌های کلیدی: دانشگاه علوم پزشکی رفسنجان، اعضای هيأت علمي، فعاليت‌هاي پژوهشي

Title: بررسی تأثیر آموزش بر اساس مدل فرانظری در میزان فعالیت جسمانی زنان خانه‌دار شهر زاهدان در سال 1390

http://hsr.mui.ac.ir/index.php/jhsr/article/view/535

مقدمه: بر اساس آمارهاي سازمان جهاني بهداشت در سال 2003، عدم فعاليت فيزيكي موجب مرگ 9/1 ميليون نفر در سطح جهان شده است. با‌ وجود فواید بی‌شمار فعالیت جسمانی، بیش از 60 درصد بالغين جهان و بيش از 80 درصد بزرگسالان ايراني از نظر فيزيكي غير فعال هستند. مطالعات کشور نشان می‌دهد، سلامت زنان در بیشتر حیطه‌ها کمتر از مردان است. همچنین زنان از بیماری‌هایی رنج می‌برند که با عدم فعالیت جسمانی مرتبط می‌باشد. مدل فرانظری به عنوان يك مدل جامع شناخته شده در زمينه ورزش است. مراحل تغییر، تعادل تصمیمی و خودکارامدی از سازه‌های اصلی این مدل هستند. هدف از انجام اين مطالعه، تعیین تأثیر آموزش بر اساس مدل فرانظری در میزان فعالیت جسمانی زنان خانه‌دار شهر زاهدان بود. روش‌ها: در مطالعه مداخله‌ای شاهددار حاضر، 220 زن خانه‌دار (محدوده سنی 52 ± 19 سال) ساکن آپارتمان در شهر زاهدان مورد بررسي قرار گرفتند که 110 نفر در گروه مداخله و 110 نفر در گروه شاهد قرار داشتند. روش نمونه‌گيري اولین بلوک به روش نمونه‌گیری تصادفی انجام گرديد، سپس به ترتیب شماره، سایر بلوک‌ها نمونه‌گیری شدند تا زمانی که نمونه‌ها کامل شد. جمع‌آوري اطلاعات از طریق پرسش‌نامه محقق ساخته صورت گرفت که اعتبار علمی آن به روش روایی محتوا و پایایی آن با روش Cronbach¢s alpha و همبستگی تعیین گردید. داده‌های جمع‌آوری شده با استفاده از نرم‌افزار SPSS نسخه 18 تجزیه و تحلیل و 05/0 > P از نظر آماري معني‌دار تلقی شد. یافته‌ها: این تحقیق نشان داد که قبل از آموزش، اختلاف معنی‌دار بین گروه‌های مورد مطالعه از نظر آگاهی، نگرش و سازه‌های مدل فرانظری وجود نداشت، اما پس از مداخله آموزشی، افزایش معنی‌داری در میانگین نمره آگاهی، نگرش و سازه‌های مدل در گروه مداخله ملاحظه گردید (001/0 > P). نتیجه‌گیری: مداخله آموزشی در انجام فعالیت جسمانی تأثیر مثبت زیادی دارد و باید در مراکز ذی‌ربط از جمله دانشگاه‌ها و مراکز تربیت بدنی، برنامه‌های عملی مناسب جهت آموزش افراد تدوین و اجرا شود.

Title: بررسی شیوع کوتاه قدی، کم‌وزنی و لاغری در کودکان روستایی زیر 6 سال زاهدان بر اساس معیارهای National Center for Health Statistic (NCHS) در سال 1388

http://hsr.mui.ac.ir/index.php/jhsr/article/view/506

مقدمه: ارزیابی رشد کودکان علاوه بر نشان دادن وضعیت تغذیه‌ای و سلامت کودکان در هر جامعه به عنوان ابزار معتبری برای سنجش کیفیت زندگی به شمار می‌رود. بر اساس آمار United Nations Children's Fund (UNICEF)، روزانه چهل هزار کودک به دلیل نامناسب بودن وضع تغذیه‌ای از بین می‌روند. مطالعه حاضر با هدف، بررسی میزان و شدت سوء تغذیه در کودکان روستایی زیر 6 سال زاهدان صورت گرفت.  روش‌ها: طی یک مطالعه مقطعی، 514 کودک روستایی 6 تا 59 ماهه به روش نمونه‌گیری تصادفی خوشه‌ای از سطح شهرستان و از طریق پرسش‌نامه و مراجعه خانه به خانه مورد بررسی قرار گرفتند. ابزار جمع‌آوری داده‌ها، پرسش‌نامه، ترازو و قدسنج بود. داده‌ها توسط نرم‌افزار Nutritional Servey و SPSS نسخه 18 تجزیه و تحلیل و با منحنی‌های National center for health statistic (NCHS) به عنوان مرجع بررسی گردید. بر طبق این معیار کم‌وزنی، لاغری و کوتاه قدی به صورت 2- £ Z-score و شدت سوء تغذیه به صورت میانه دور بازو (Mid upper arm circumference یا MUAC) کمتر از 115 تعریف شده است. یافته‌ها: از بین کودکان بررسی شده 3/17، 5/25 و 8/6 درصد به ترتیب دچار کم‌وزنی، کوتاه قدی و لاغری بودند. میانگین قد (4/11 ± 83 در مقابل 2/12 ± 82 سانتی‌متر)، وزن (6/2 ± 11 در مقابل 6/2 ± 10 کیلوگرم) و میانه دور بازو (3/14 ± 145 در مقابل 2/16 ± 141 میلی‌متر) در مجموع در پسران بالاتر از دختران بود و به طور کلی شیوع کوتاه قدی (7/29 در مقابل 5/22 درصد)، کم‌وزنی (7/20 در مقابل 7/15 درصد)و لاغری (8/9 در مقابل 4/4 درصد) در پسران بیشتر از دختران بود (05/0 < P). نتیجه‌گیری: سوء تغذیه یکی از مشکلات عمده تغذیه‌ای در کودکان روستایی زیر 6 سال زاهدان می‌باشد. بنابراین طراحی و اجرای مداخلات مناسب برای بهبود وضع تغذیه کودکان و همچنین توجه به نقش پایش رشد در ارزیابی وضع تغذیه کودکان ضروری به نظر می‌رسد.

Title: مطالعه مقایسه‌ایی از اثر تخمیر بر قابلیت آنتی‌اکسیدانی شیر و شیر سویای حرارت دیده

http://hsr.mui.ac.ir/index.php/jhsr/article/view/591

مقدمه: بر اساس تحقیقات موجود، تخمیر شیر و شیر سویا با باکتری‌های لاکتیک به افزایش قابلیت آنتی‌اکسیدانی آن‌ها می‌انجامد، در مطالعه قبلی ما، تخمیر شیر و شیر سویای فرادما با یک سویه بومی از لاکتو باسیلوس پلنتارم قابلیت آنتی‌اکسیدانی در شیر سویای فرادما را کاهش داد. بنابراین بررسی اثر سویه باکتریایی و شدت فرایند حرارتی متحمل شده بر نمونه‌های استریلیزه و پاستوریزه غیر تخمیری و تخمیر شده شیر و شیر سویا در تحقیق حاضر مورد پژوهش قرار گرفت.  روش‌ها: بررسی افزایش قابلیت آنتی‌اکسیدانی به روش اثر مهار کنندگی رادیکال آزاد 1, 1-diphenyl-2-picrylhydrazyl (DPPHl) تخمیر با سه سویه متفاوت از لاکتوباسیلوس لاکتوباسیلوس پلانتاروم، لاکتوباسیلوس اسیدوفیلوس و لاکتوباسیلوس رامنوسوس انجام شد. یافته‌ها: اثر مهار کنندگی رادیکال آزاد DPPH در نمونه‌های (پاستوریزه و استریلیزه) شیر سویا و شیر گاو به ترتیب (67/6 ± 84/31، 99/0 ± 93/35) و (34/2 ± 99/14، 41/2 ± 24/15) حاصل شد. شاخص فوق طی تخمیر با سویه‌های مختلف مستقل از سویه باکتریایی، در شیر سویای پاستوریزه و استریلیزه به ترتیب 50/3 ± 04/45 و 21/12 ± 36/24 و در نمونه‌های مشابه شیر گاو به ترتیب مقادیر 24/5 ± 10/25 و 01/4 ± 38/24 به دست آمد. نتیجه‌گیری: تخمیر شیر سویای پاستوریزه و استریلیزه به ترتیب منجر به افزایش و کاهش قابلیت آنتی‌اکسیدانی آن می‌شود. در صورتی که در مورد نمونه‌های تخمیری شیر، قابلیت آنتی‌اکسیدانی به طور مستقل از نوع فرایند حرارتی به کار رفته سبب افزایش قابلیت فوق می‌گردد.

Title: بررسی اثرات مواجهه با صدا بر میزان هورمون کورتیزول سرم و برخی پارامترهای خونی کارگران صنعت فولاد

http://hsr.mui.ac.ir/index.php/jhsr/article/view/645

مقدمه: صدا یکی از مهم‌ترین عوامل فیزیکی زیان‌آور محیط‌های صنعتی به شمار می‌آید. صدا دارای اثرات شنوایی و غیر شنوایی بوده است که از اثرات مهم آن بر سلامتی می‌توان به آسیب دستگاه شنوایی، اثر روی اندام بینایی، اثر بر سیستم تعادلی، اثرات عصبی و روانی، اثر روی الکترولیت‌ها، اثرات فیزیولوژیکی و اثرات ذهنی اشاره کرد. به دلیل اهمیت موضوع، این مطالعه با هدف تعیین اثرات مواجهه با صدا بر میزان هورمون کورتیزول سرم و برخی پارامترهای خونی کارگران صنعت فولاد انجام شد.  روش‌ها: در این مطالعه مقطعی، تعداد 50 نفر از بین شاغلین صنعت فولاد به عنوان گروه مورد مطالعه شرکت کردند. ابتدا جهت گردآوری اطلاعات دموگرافیک برای هر فرد، پرسش‌نامه‌ای تکمیل شد. جهت بررسی پارامترهای خونی نظیر تغییرات هورمون کورتیزول، چربی خون و قند خون قبل و بعد از شیفت کاری از افراد شرکت کننده نمونه خون گرفته شد. شاخص LAeq به وسیله دستگاه صداسنج CEL مدل 440 در محل توقف کارگران اندازه‌گیری و صدای دستگاه در فرکانس‌های اکتاوباند آنالیز شد. مطالعه در دو مرحله میدانی و آزمایشگاهی صورت گرفت. در مرحله آزمایشگاهی، کارگران به مدت 5 دقیقه به ترتیب با تراز صدای 85، 95 و 105 دسی‌بل در سه روز متوالی مواجه شدند و در نهایت نتایج به دست آمده از آزمایش خون از طریق نرم‌افزار SPSS تجزیه و تحلیل شد. یافته‌ها: یافته‌های آزمایشگاهی نشان داد که میزان هورمون کورتیزول بعد از مواجهه با صدا، در هر سه تراز صوت 85، 95 و 105 دسی‌بل افزایش داشت، اما این افزایش از نظر آماری معنی‌دار نبود (05/0 < P). چربی خون (TG يا Triglyceride) نیز بعد از مواجهه در هر سه تراز کاهش داشت که این تغییرات نیز به لحاظ آماری معنی‌دار نبود (05/0 < P). میزان  High density lopoprotein (HDL) و Low density lipoprotein (LDL) نیز در تراز 85 دسی‌بل روند کاهشی داشت، اما نسبت به حالت قبل از مواجهه اختلاف معنی‌داری نداشت (05/0< P). میزان قند خون افراد نیز در هر سه تراز افزایش داشت که این افزایش در تراز 95 دسی‌بل معنی‌دار بود (001/0 > P). نتیجه‌گیری: نتایج مطالعه حاضر نشان داد که مواجهه با صدا در ترازهای بالا موجب تغییر در پارامترهای فیزیولوژیک نظیر هورمون کورتیزول سرم، چربی و قند خون می‌شود اما با این وجود، به جز پارامتر قند خون که در تراز 95 دسی‌بل اختلاف معنی‌داری نسبت به حالت قبل از مواجهه داشت، به دلیل معنی‌دار نبودن تغییرات سایر پارامترها، نمی‌توان صدا را به عنوان عامل مؤثر بر پارامترهای کلینیکی معرفی نمود.

Title: ارایه الگوی ارزیابی ریسک‌های بهداشت حرفه‌ای و محیط در حمل و نقل فرآورده‌های نفتی با استفاده از تلفیق روش Failure mode and effect analysis (FMEA) با Analytical hierarchy process (AHP)

http://hsr.mui.ac.ir/index.php/jhsr/article/view/754

مقدمه: یکی از مهم‌ترین خطرات موجود در حمل و نقل مواد خطرناک، تصادفات جاده‌ای است که می‌تواند اثرات مخربی را بر محیط و ایمنی جاده‌ها داشته باشد. با استفاده از تکنیک‌های مدیریت ریسک می‌توان عوامل ایجاد حادثه را شناسایی و کنترل نمود.  روش‌ها: در ابتدا تمام عوامل مؤثر در ریسک محیط زیست ناشی از حمل و نقل فرآورده‌های نفتی از انبار نفت تا مقصد تعیین شد و با استفاده از روش تصمیم‌گیری سلسله مراتبی (AHP يا Analytical hierarchy process) وزن‌دهی گردید و مدل مفهومی آن تعریف شد. در قدم بعدی با استفاده از مدل به دست آمده بر اساس منطق Failure mode and effect analysis (FMEA) مدل نهایی تعیین گردید. با استفاده از مدل نهایی به دست آمده، مسیر حمل مورد ارزیابی قرار گرفته شد. یافته‌ها: میزان ریسک برای مناطق مختلف تردد تانکرهای نفتکش به جز دو منطقه‌ای که در حومه شهر و یا در داخل شهر بوده است و کنترل پلیس ترافیکی به حداقل می‌رسد قابل قبول می‌باشد. علت بالا بودن میزان ریسک در این مناطق، وجود منطقه مسکونی در این محدوده‌ها، وجود گردنه و مسیرهای با شیب زیاد، مجاورت با مناطق کشاورزی و عدم کنترل دقیق توسط پلیس راه می‌باشد. نتیجه‌گیری: انتقال انبارهای نفت از داخل شهر به خارج از شهر و کنترل دقیق و سختگیرانه، صلاحیت راننده و تانکر حمل فرآورده نفت توسط پلیس و آموزش و آشنا‌سازی پلیس با اصول ممیزی ایمنی تانکرها از مواردی است که می‌تواند در کاهش میزان ریسک آلودگی‌های محیط زیست ناشی از حمل فرآورده‌های نفتی توسط تانکرها به آن اشاره نمود.

Title: اثر بخشی برنامه مداخله ای بر مبنای مدل فرانظری در ارتقاء فعالیت بدنی منظم در کارکنان اداری

http://hsr.mui.ac.ir/index.php/jhsr/article/view/798

مقدمه: با وجود نقش مهم فعالیت بدنی در سلامتی، رفاه و کیفیت زندگی، امروزه اکثر افراد از فعالیت بدنی مناسبی برخوردار نمی‌باشند. هدف از انجام اين مطالعه، بررسی اثربخشی مداخله آموزشی بر مبنای مدل فرانظری در ارتقای فعالیت بدنی منظم کارکنان اداری دانشگاه علوم پزشکی ایلام در سال 91-1390 بود. روش‌ها: مطالعه حاضر یک پژوهش مداخله‌ای (Interventional) از نوع قبل و بعد بود كه بر روي 97 نفر از کارکنان دانشگاه علوم پزشکی ایلام که به روش نمونه‌گیری تصادفی انتخاب شده بودند، انجام گرفت. شرکت کنندگان ابتدا پرسش‌نامه سنجش متغیرهای دموگرافیکی و‌ سازه‌های تئوری فرانظری را به صورت خودگزارشی تکمیل نمودند. سپس محتوای آموزشي طراحی شده بر اساس تئوری فرانظری را در قالب جزوه، کتابچه و سی‌دی آموزشی انجام فعالیت بدنی دریافت نمودند. پس از 3 ماه و به منظور تعیین اثربخشی مداخله، پرسش‌نامه سنجش سازه‌های مدل فرانظری دوباره تکمیل گردید. داده‌های مطالعه با استفاده از نرم‌افزار آماری SPSS نسخه 16 و آزمون‌های آماری Fisher exact test، Paired t و ANOVA مورد تجزیه و تحلیل قرار گرفت. یافته‌ها: ميانگين سنی شرکت کنندگان در مطالعه 73/6 ± 16/32 سال بود که 42 درصد مذکر و 55 درصد مؤنث بودند. از لحاظ نوع ورزش انتخابی 3/42 درصد پیاده‌روی، 6/52 درصد شنای تفریحی، 1/3 درصد ورزش‌های بدنسازی و 2/1 درصد کوهنوردی را برای انجام فعالیت بدنی منظم انتخاب نمودند. یافته‌های حاصل از مقایسه مراحل تغییر انجام فعالیت بدنی نشان داد که مداخله آموزشی باعث پیشرفت معنی‌دار در مراحل تغییر شرکت کنندگان شده است (05/0 > P). همچنین مقایسه میانگین سازه‌های تعادل در تصمیم‌گیری، فرایندهای تغییر و خودکارامدی انجام فعالیت‌های ورزشی نشان دهنده افزایش معنی‌دار میانگین سازه‌های مدل فرانظری در مقایسه بعد از مداخله بود (05/0 > P). یافته‌های پژوهش حاکی از افزایش معنی‌دار میانگین سطوح آگاهی، تسکین نمایشی، شرطی‌سازی تقابلی، کنترل محرک، روابط یاری دهنده و خودآزادی بعد از مداخله در مقایسه با قبل از آن بود (05/0 > P)، اما افزایش میانگین در سایر سطوح فرایندهای شناختی و رفتاری معنی‌دار نبود. نتیجه‌گیری: بر اساس نتایج به دست آمده، طراحی برنامه‌های مداخله‌ای بر مبنای مدل‌های تغییر می‌تواند باعث ارتقای رفتارهای مرتبط با سلامت گردد.

Title: مقایسه مقدار نیترات در سبزیجات آبیاری شده با آب زاینده‌رود و آب چاه

http://hsr.mui.ac.ir/index.php/jhsr/article/view/1201

مقدمه: دریافت زیاد نیتریت و نیترات، اثرات نامطلوبی بر سلامتی انسان دارد، از جمله عوارض آن بیماری متهموگلوبینمیا در کودکان و سرطان‌زایی در بزرگسالان را می‌توان نام برد. سبزیجات از منابع اصلی نیتریت و نیترات در رژیم غذایی محسوب می‌شوند. این مطالعه جهت بررسی تأثیر نوع آب استفاده شده برای آبیاری بر مقدار نیترات سبزیجات انجام شد. روش‌ها: از منطقه شرق اصفهان تعداد 106 نمونه از 8 نوع سبزی پر مصرف (خیار، گوجه، کاهو، پیاز، تره، جعفری، اسفناج، سیب‌ زمینی) آبیاری شده با آب زاینده‌رود و 106 نمونه از همان سبزیجات که با چاه آبیاری شده بود به طور تصادفی جمع‌آوری گردید. پس از آماده‌سازی نمونه‌ها مقدار نیترات و نیتریت به روش مرجع اسپکتروفتومتری اندازه‌گیری شد و داده‌ها با استفاده از نرم‌افزار SPSS مورد تجزیه و تحلیل قرار گرفت. یافته‌ها: میانگین و انحراف معیار نیترات در خیار، گوجه، پیاز، تره، کاهو، جعفری، اسفناج، سیب زمینی آبیاری شده با آب زاینده‌رود به ترتیب 1/1 ± 30، 4/1 ± 66/30، 59/2 ± 25/45، 35/4 ± 66/230، 28/19 ± 8/173، 44/8 ± 66/319، 47/18 ± 25/321 و 2 ± 25/64 میلی‌گرم به ازای کیلوگرم وزن مرطوب و در خیار، گوجه، پیاز، تره، کاهو، جعفری، اسفناج، سیب زمینی آبیاری شده با آب چاه به ترتیب 91/0 ± 73/26، 77/2 ± 66/26، 87/2 ± 41/28، 56/20 ± 83/161، 2/58 ± 71/275، 38/37 ± 5/312، 67/28 ± 311 و 06/2 ± 9/116 میلی‌گرم به ازای کیلوگرم وزن مرطوب بود. مقدار نیتریت به علت ناچیز بودن مقدار آن، قابل اندازه‌گیری نبود. آزمون t مستقل نشان داد که میانگین نیترات در خیار، گوجه، پیاز و تره آبیاری شده با آب زاینده‌رود به طور معنی‌داری بیشتر از سبزیجات آبیاری شده با آب چاه بود (001/0 = P) و میانگین نیترات در جعفری و اسفناج تفاوت معنی‌داری نداشت. در ضمن میانگین نیترات سیب زمینی و کاهو آبیاری شده با آب چاه به طور معنی‌داری بیشتر از سیب زمینی و کاهو آبیاری شده با آب چاه بود. نتیجه‌گیری: میانگین نیترات در سبزیجات مختلف یکسان نیست. خیار و گوجه کمترین میزان و جعفری و اسفناج بیشترین مقدار را در میان نمونه‌های مورد بررسی داشتند و در اکثر موارد مقدار نیترات در سبزیجات آبیاری شده با آب رودخانه بیشتر است که می‌تواند به دلیل ورود فاضلاب‌های صنعتی به آب رودخانه باشد که توجه خاص مسوؤلین امر را می‌طلبد.

Title: بررسی تأثیر بسته آموزشي كنترل مالاريا بر میزان مهارت‌هاي شناختي و عملکردي پزشكان استان سیستان و بلوچستان در سال 1388

http://hsr.mui.ac.ir/index.php/jhsr/article/view/1202

مقدمه: مالاريا يكي از 3 بيماري مهمي است كه در سراسر جهان مطرح است. 65 درصد موارد مالاریا در ایران از استان سیستان و بلوچستان گزارش می‌شود. پزشکان نقش مهمی در کنترل بیماری در منطقه به عهده دارند. این مطالعه تأثیر بسته آموزشی کنترل مالاریا را بر روی مهارت‌های شناختی و عملکردی پزشکان در منطقه بررسی می‌نماید. روش‌ها: یک مطالعه مداخله‌ای به صورت قبل و بعد، بدون گروه شاهد، اثر بسته آموزشي كنترل مالاريا (شامل 4 کتابچه و یک سی‌دی آموزشی) را بر میزان مهارت‌های شناختی و عملکردی پزشکان در سال 1388 بررسی نمود. 30 پزشك شاغل در مراکز بهداشتی درمانی از 4 شهرستان چابهار، نیکشهر، ایرانشهر و سرباز مورد مطالعه قرار گرفتند. ابزار جمع‌آوری اطلاعات در این مطالعه، پرسش‌نامه (شامل 62 سؤال تشریحی) و چک لیست (برای سنجش 13 مهارت) بود. نتایج این مطالعه با استفاده از نرم‌افزار SPSS و آزمون‌های آماری ناپارامتریک و Paired- t مورد تجزیه و تحلیل قرار گرفت. یافته‌ها: میزان مهارت شناختی پزشکان از 8/13 ± 28 (قبل از مداخله) به 4/15 ± 8/74 (بعد از مداخله) افزایش یافته است که این افزایش از نظر آماری (0001/0 > P) معنی‌دار می‌باشد. میزان مهارت عملکردی پزشکان از 3/10 ± 5/30 (قبل از مداخله) به 3/11 ± 6/51 (بعد از مداخله) افزایش یافته است که این افزایش از نظر آماری (0001/0 > P) معنی‌دار می‌باشد. نتیجه‌گیری: نتایج مطالعه نشان داد که ارایه یک بسته آموزشی که شامل کتابچه و سی‌دی آموزشی است، روشی مناسب، ساده و کاربردی برای افزایش مهارت‌های شناختی و عملکردی پزشکان می‌باشد.
